# Inborn errors of immunity with atopic phenotypes in the allergy and immunology clinic: a practical review

**DOI:** 10.1097/ACI.0000000000001059

**Published:** 2025-02-13

**Authors:** Ivan Taietti, Francesco Catamerò, Lorenzo Lodi, Mattia Giovannini, Riccardo Castagnoli

**Affiliations:** a Pediatric Unit, Department of Clinical, Surgical, Diagnostic, and Pediatric Sciences, University of Pavia; b Pediatric Clinic, Fondazione IRCCS Policlinico San Matteo, Pavia; c Department of Health Sciences, University of Florence; d Immunology Unit, Meyer Children's Hospital IRCCS; e Allergy Unit, Meyer Children's Hospital IRCCS, Florence, Italy

**Keywords:** allergy, atopy, inborn errors of immunity, inborn errors of immunity with atopic phenotypes, precision medicine, primary immunodeficiencies

## Abstract

**Purpose of review:**

Inborn errors of immunity with atopic phenotypes (IEIwA) are a subgroup of IEI that may present with severe and/or multiple atopic clinical manifestations. Because of their specific clinical management and prognosis, it is important to distinguish IEIwA from multifactorial allergic diseases. We aimed to review the main clinical manifestations associated with IEIwA and summarize the available data regarding the precision medicine approach for these conditions.

**Recent findings:**

IEIwA include more than 50 monogenic disorders marked by different immune dysregulation mechanisms such as alterations in cytokine signaling, T cell receptor function, mast cell activation, and skin barrier integrity. A critical role in diagnosis is played by advanced genetic testing. Emerging treatments include targeted monoclonal antibodies and small molecules, whereas hematopoietic stem cell transplantation (HSCT) is still a valid option for some specific disorders and may be curative also on atopic manifestations.

**Summary:**

The recognition and accurate diagnosis of IEIwA are crucial for timely and appropriate therapeutic intervention. The diagnosis should be suspected according to the presence of ‘red flags’ at clinical evaluation stage, such as early-onset severe atopy, recurrent/atypical infections, and autoimmunity. The diagnostic confirmation requires genetic testing. Precision medicine approaches like biological therapies and HSCT seem to provide promising results. It is worth noting that clinical and translational research in the field of IEIwA is currently paving the way for a more thorough understanding of the molecular bases of common allergic diseases.

## INTRODUCTION

According to an increasing amount of evidence, immune dysregulation, including severe and multiple atopic clinical manifestations, might be the main clinical feature of specific inborn errors of immunity (IEI) [1,2,3^▪^,^4^]. Inborn errors of immunity with atopic phenotypes (IEIwA) can be defined as a diverse and complex group of human monogenic IEI that, among their clinical manifestations, may present with severe allergic and atopic (type 2) effector–related signs and symptoms [5]. Recently, Lyons and Milner referred to these conditions as primary atopic disorders (PAD) [5]. The different clinical manifestations may be related to chronic type 2 T-helper (Th2) and eosinophil-mediated allergic inflammation, abnormal mast cell activation, and exuberant immunoglobulin E (IgE) production [5]. The latter might be caused by alterations in cytokine signaling, T cell receptor (TCR) signaling and actin dynamics, tolerance mechanisms, intrinsic mast cell functions, or skin barrier [6].

It is worth mentioning how several clinical features in IEIwA overlap with those noticed in common multifactorial atopic disorders. Recognizing IEI in the context of an allergic phenotype is crucial to ensure quick diagnosis and adequate therapy. The clinical management and expected outcomes for IEIwA are not the same as in typical allergic conditions. Moreover, IEIwA currently represents an active research field in clinical immunology, especially concerning the identification of the basic pathogenetic mechanisms. The latter is crucial for both the precision medicine approach for these conditions and to provide insights into the treatment of more common multifactorial allergic diseases [7–10]. 

**Box 1:**
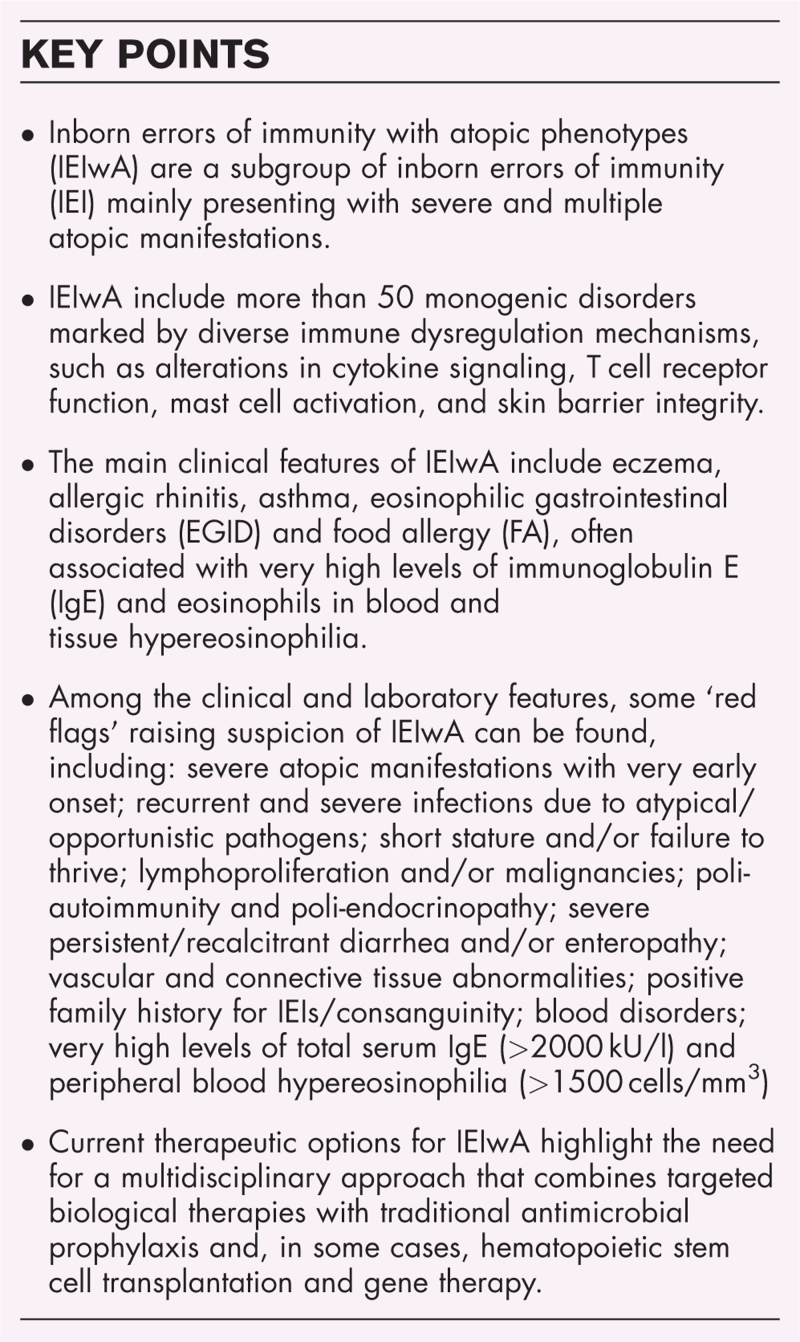
no caption available

## CLINICAL MANIFESTATIONS

It has been estimated that IEIwA cover over 50 monogenic disorders [11^▪▪^]. As hypothesized by some authors, other possible exogenous factors influencing the clinical phenotype and the age of presentation could be environmental, including diet, infections and the microbiota- host interactions [8,12].

Atopy can develop through different pathways which mainly vary from specific defects in immune cells and epithelial barrier function to metabolic alterations [5]. Different clinical phenotypes may be sustained by gain-of-function (GOF) or loss-of-function (LOF) mutations in the same gene. Moreover, the causes of the phenotypic variability in IEI can also depend on different activity levels of mutant proteins related to hypomorphic and hypermorphic mutations [2].

Common clinical features of IEIwA include, among others, eczema, allergic rhinitis, asthma, eosinophilic gastrointestinal disorders (EGID) and food allergy (FA), often associated with considerably high levels of IgE and eosinophils in blood and tissue hypereosinophilia. The number and complexity of atopic phenotypes described is constantly increasing [13,14].

To simplify the discernment of monogenic IEIwA from polygenic disorders for clinicians, first we have grouped IEIwA based on the dominating pattern of clinical manifestations [15]: (1) hyper-IgE syndromes (HIES); (2) Wiskott–Aldrich syndrome (WAS) and WAS-like (WASL) conditions; (3) immune dysregulation, polyendocrinopathy, enteropathy, X-linked (IPEX) and IPEX-like conditions; (4) caspase Recruitment Domain (CARD) Family Member 11 (CARD11) – B cell chronic lymphocytic leukemia/lymphoma 10 (BCL10) – mucosa-associated lymphoid tissue lymphoma translocation gene 1 (MALT1) (CBM-opathies); (5) miscellaneous.

### Hyper-immunoglobulin E syndromes

HIES include different clinical entities characterized by increased serum IgE and wide variable clinical spectrum. Signal transducer and activator of transcription (STAT) 3 (*STAT3*)-autosomal dominant (AD)-HIES (also known as Job Syndrome) is characterized by eczema, skin abscesses, chronic mucocutaneous candidiasis (CMC), recurrent pneumonia leading to pneumatoceles, and skeletal and connective tissue abnormalities (e.g., bone fragility, scoliosis, decidual teeth retention, vascular abnormalities). Several factors, such as early-onset eczema, peculiar thickened texture of the facial skin, retro auricular fissures, severe folliculitis of the axillae and groin, cold abscesses (due to poor inflammation), distinctive facial and skeletal features, and low frequency of allergy, differentiate it from common atopic dermatitis [15–17]. Despite some cases of negativity of specific IgE values and skin prick testing, as well as eosinophilia, elevated serum IgE levels (often higher than 2000 IU/ml) are typical; conversely, immunoglobulin levels are usually normal, with possible impairment of specific antibody responses to encapsulated bacteria. Moreover, several studies have reported memory T and B cells reduction as well as very low interleukin (IL)-17 producing T cells [18–20].

A similar phenotype, mostly overlapping with AD-HIES, was found in patients with biallelic mutations in zinc finger protein 341 (*ZNF341*) or with autosomal recessive (AR) mutations of the IL6 signal transducer (*IL6ST*) gene. More recently, AD mutations in *IL6ST* have been identified as the second genetic cause of AD-HIES. Likewise, AR IL6 receptor (*IL6R*) deficiency manifests partial overlapping with AD-HIES (no skeletal abnormalities) [16,21].

LOF mutations in Dedicator of cytokinesis 8 (*DOCK8*) can cause a combined immunodeficiency that is noteworthy for severe viral skin infection [e.g., Human Papillomavirus (HPV), molluscum contagiosum, Herpes Simplex Virus (HSV) 1 and 2] – increasing risk for malignancies (e.g. HPV-associated squamous cell carcinomas, rapidly progressive T-cell lymphoma) – in addition to severe bacterial respiratory sinopulmonary infection. The prevalence and severity of allergic disease (e.g., anaphylaxis) in *DOCK8* deficiency is considerable due to their highest ratio of food-specific IgE to total IgE, while in STAT3-HIES it is yet unspecific [6,19,22]. Severe eczema is quite common, especially in association with warts, and some cases of autoimmunity have been reported [15–20].

Tyrosine kinase 2 (*TYK2*) deficiency can be found in case of HIES characterized by severe dermatitis and a peculiar susceptibility to intracellular bacteria, for example, (mycobacteria, *Salmonella*) and viruses [23].

Several phenotypes with significant overlap between allergic and connective tissue are now known. ERBB2-interacting (*ERBIN*) protein deficiency is characterized by eczema, eosinophilic esophagitis (EoE), recurrent respiratory infections, high IgE levels as well as skeletal and connective tissue abnormalities (i.e., joint hypermobility and vascular abnormalities) [24]. Also, Loeys–Dietz syndrome [AD Transforming growth factor beta receptor I (*TGFBR*) deficiency] shows a Marfan-like syndrome with noticeable prevalence of allergic diseases [15].

Moreover, as in the case of the Comèl–Netherton syndrome [AR serine peptidase inhibitor Kazal type 5 (*SPINK5*) defect], HIES may be related to a skin barrier disruption showing congenital ichthyosis, bamboo hair, atopic diathesis, increased bacterial infections with impaired responses to polysaccharide vaccination, enteropathy, and failure to thrive [25].

Metabolic disturbances can cause HIES, too. In fact, phosphoglucomutase 3 (*PGM3*) deficiency induces an AR disease with immunodeficiency and tendency to bone marrow failure, severe atopy, severe skeletal dysplasia, and neurodevelopmental delay [26].

Finally, GOF AD mutations in *STAT6* have been recently reported and are now considered the prototype of IEIwA. Indeed, GOF mutations in *STAT6* lead to a syndrome that includes: (i) severe early-onset atopic disease in all patients (recalcitrant eczematous dermatitis, food or drug allergies, asthma, and severe or recurrent anaphylaxis), with particularly elevated serum IgE levels and hypereosinophilia; (ii) gastrointestinal signs and symptoms (75%) (severe eosinophilic gastrointestinal inflammation and, occasionally, diarrhea or protein-losing enteropathy) (iii) mild recurrent skin and respiratory infections (50%); (iv) skeletal abnormalities similar to STAT3 AD-HIES (osteoporosis, pathological fractures, joint hyperextensibility, and/or vascular abnormalities) (33%); (v) short stature (40%); (vi) significant lymphadenopathy, large polypoid intestinal nodules, and recurrent B-cell lymphomas (e.g., follicular lymphoma) [27,28^▪▪^,^29^^▪▪^].

### Wiskott–Aldrich syndrome and Wiskott–Aldrich syndrome-like conditions

LOF in X-linked (XL) WAS protein (*WASP*) leads to WAS, usually characterized by severe atopic dermatitis (eczema), thrombocytopenia (with characteristic bloody diarrhea) with small platelets (mean platelet volume, MPV < 6 fl), immunodeficiency/immunedysregulation with recurrent bacterial and/or chronic viral (e.g., herpesviruses, papillomavirus, and molluscum contagiosum) infections, autoimmunity (e.g., inflammatory bowel diseases, hemolytic anemia, arthritis, and IgA nephropathy), allergy, and neoplastic disease [mostly hematological: e.g., Ebstein–Barr virus (EBV) driven lymphoproliferative disease, lymphoma and leukemia]. WASL phenotypes (thrombocytopenia with or without small platelets), including substantial atopic diathesis, can be caused by biallelic LOF AR mutations in WAS/WASL interacting protein family member 1 (*WIPF1*) encoding WASP-interacting protein (*WIP*) and actin-related protein 2/3 complex subunit 1B (*ARPC1B*) [6,15]. Moreover, AR Actin Related Protein 2/3 Complex Subunit 1B (*ARPC1B*)-deficiency [30] and mutation in Cell division cycle 42 (*CDC42*) [31] show, respectively, WAS-like phenotype with mild thrombocytopenia and neonatal-onset cytopenia, autoinflammation, rash, as well as episodes of hemophagocytic lymphohistiocytosis (NOCARH) and neurodevelopmental delay.

### Immune dysregulation, polyendocrinopathy, enteropathy, X-linked and immune dysregulation, polyendocrinopathy, enteropathy, X-linked-like conditions

IPEX [XL forkhead box P3 (*FOXP3*) mutations] and IPEX-like syndromes [AR Cluster of Differentiation 25 (*CD25*) deficiency, AR/AD *STAT5b* deficiency, AD *STAT1* GOF, AR Itchy E3 Ubiquitin Protein Ligase (*ITCH*) deficiency] are included in the category of Treg-opathies, namely disorders caused by genetic mutations lead to defects in molecules essential for the development, survival, and/or function of regulatory T (Treg) cells [3^▪^,^15^]. The first one tends to appear in early age, usually in the form of severe eczematous dermatitis, polyendocrinopathy (e.g., early onset/neonatal type 1 diabetes mellitus and/or autoimmune thyroiditis) and enteropathy. Such condition often requires parenteral nutrition, with different immune dysregulation features (cytopenia with hemolytic anemia and thrombocytopenia, autoimmune hepatitis, nephropathy, and myopathy) [32]. The disease course may worsen due to life-threatening or even fatal bacteria, viruses or fungi infections [33]. Several clinical features can help identify the different IPEX-like phenotypes. (i) AR CD25 deficiency due to mutation in Interleukin 2 Receptor Subunit Alpha (*IL2RA*): bullous pemphigoid, higher susceptibility to infection with chronic viral, fungal and bacterial infections, lymphadenopathy, hepatosplenomegaly and polyautoimmunity [34]; (ii) AR *STAT5b* deficiency: growth-hormone insensitive dwarfism and dysmorphic features. AD *STAT5b* deficiency: growth-failure and eczema without immune defects; (iii) AD *STAT1* GOF CMC and autoimmunity; (iv) AR *ITCH* deficiency: autoimmunity, dysmorphic facial features and neurodevelopmental delay [15].

### CMB-opathies

This group of IEIwA encompasses different and heterogeneous clinical entities. Complete biallelic loss of *CARD11* signaling causes profound combined immunodeficiency (CID) requiring Hematopoietic stem cell transplantation (HSCT) [6]; instead, heterozygous dominant negative mutations are related to *CARD11*-associated atopy with dominant interference of NF-κB signaling (CADINS) [35]. CADINS appears with severe atopic disease (e.g., atopic dermatitis, rhinoconjunctivitis, food allergy, EoE, and/or asthma), respiratory tract infections and cutaneous viral infections, as well as immune dysregulation with Th-2 skewed immune response with increased IgE and eosinophilia, normal/low B-cell numbers, normal absolute T- and NK-cell numbers, impaired T-cell proliferation, and possible hypogammaglobulinemia with altered response of the specific antibody [36]. AD *CARD14* deficiency causes a CADINS-like phenotype characterized by recurrent pyogenic infections [37].

Moreover, AR *MALT1* LOF mutation leads to CID with severe atopic dermatitis, inflammatory gut disease, elevated serum IgE and other allergic phenotypes. Frequent viral skin infections, as well as bacterial respiratory and gastrointestinal tract infections, failure to thrive and periodontal disease are also common clinical manifestations [38].

### Miscellaneous

Omenn syndrome (OS) characterized by erythroderma or neonatal eczematous rash, lymphadenopathy, eosinophilia, and combined immune deficiency [15], is not considered as typical allergy, although being characterized by Th2 systemic inflammation. In fact, allergen-specificity is unlikely to be included among the effector cells. Also, skin lesions tend to be more severe and earlier in onset compared to those associated with common atopic dermatitis [39]. However, these lesions have many pathological features in common with atopic dermatitis and can be clinically indistinguishable from many acquired (e.g., infections, drug hypersensitivity reactions, inflammatory skin disorders) and inherited (e.g., inborn errors of metabolism, ichthyoses) infantile erythrodermas. OS is associated with multiple genetic abnormalities, both severe combined immunodeficiency (SCID)-associated genes, and not (e.g., *RAG1, RAG2, IL2RG, IL7R, LIG4, ADA, DCLRE1C, RMRP, CHD7, ZAP70*, 22q11del) [5].

AR LOF mutations in capping protein regulator and myosin 1 linker 2 (*CARMIL2*) can cause CID associated with severe atopic dermatitis, elevated IgE, allergic asthma, food allergy, cold urticaria, recurrent bacterial and fungal infections – including invasive tuberculosis and mucocutaneous candidiasis – EBV lymphoproliferation and other malignancies [40]. AD Janus Kinase 1 (*JAK1*) GOF mutations lead to hypereosinophilic syndrome with severe eosinophilia, eosinophilic enteritis, hepatosplenomegaly, autoimmune thyroiditis, poor growth, and viral infections [41]. Hypereosinophilic syndrome can also appear as a consequence of somatic GOF mutations in *STAT5b*, resembling a phenocopy of IEI marked by nonclonal eosinophilia, atopic dermatitis, urticarial rash and diarrhea [42].

## DIAGNOSTIC APPROACH

Patients with IEIwA typically have unique associated clinical manifestations and laboratory results needing careful analysis to identify the underlying disease. Atopic manifestations are often severe and tend to appear with a frequent early-onset atopic disease in pediatric age (i.e., at birth or in the first months of life). In addition, standard therapy is often ineffective, so targeted therapies become necessary: severe and recalcitrant eczema, severe asthma, multiple food allergies, anaphylaxis, unresponsive eosinophilic gastrointestinal disease are typical. Several clinical features, such as congenital ichthyosis, skeletal abnormalities, neurodevelopmental delay, diarrhea, endocrinopathy, bleeding, and/or failure to thrive, may be associated with IEIwA. Recurrent/severe infections (especially due to opportunistic pathogens and *Herpesviridae*, including cytomegalovirus (CMV), BV, and HHV-6) are generally possible as well [13,15].

Checking for a family history of IEIwA, consanguinity, and any pre and perinatal factors that may have affected the early development of the immune system, including maternal infections during pregnancy, becomes therefore fundamental [15].

Based on the available evidence, high Th2 biomarkers and associated immunological abnormalities are crucial in the diagnostic work-up [7,13,15,43,44]. The list of laboratory tests that could be helpful in case of suspected patients includes:

(1) Complete blood with differential count (CBC);
(2) Serum immunoglobulin (Ig) [IgA, IgM, IgG, IgE], and IgG subclasses quantitative determination;
(3) Extended T and B cell immunophenotype (including Treg, Th17, T follicular cells, HLA-DR expression);
(4) Specific antibody levels against recall antigens (vaccines, group ABO, others);
(5) Allergy testing: skin prick test and/or allergen-specific IgE measurement;
(6) Markers of organ-specific and tissue-specific autoimmunity.
(7) Disease-specific flow cytometry tests.

**FIGURE 1:**
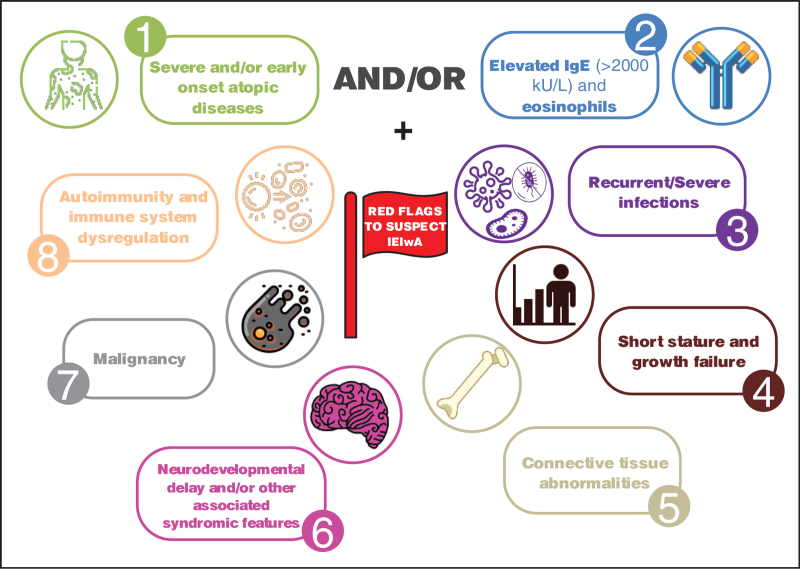
Summary of the red flags to suspect inborn errors of immunity with atopic phenotypes (IEIwA). Created with icons8.com.

Moreover, ‘red flags’ [13,15] (Fig. 1) can be identified among clinical and laboratory features, as they rise suspicion of possible IEIwA:

(1) severe atopic manifestations (especially when multiple) with very early onset (and unresponsiveness to standard treatment);
(2) recurrent and severe infections due to atypical/opportunistic pathogens;
(3) short stature and/or failure to thrive;
(4) lymphoproliferation and/or malignancies;
(5) poli-autoimmunity;
(6) severe persistent/recalcitrant diarrhea (even with eosinophilic infiltration) and/or enteropathy;
(7) poli-endocrinopathy (with particular regard to early-onset, eventually neonatal, diabetes mellitus type-1);
(8) vascular and connective tissue abnormalities;
(9) dysmorphic features;
(10) positive family history for IEIs/consanguinity;
(11) blood disorders (i.e., purpura, cytopenia);
(12) very high levels of total serum IgE (>2000 kU/l);
(13) peripheral blood hypereosinophilia (>1.500 cells/mm^3^)

Because of such warning signs, diagnostic genetic testing, including gene panels with next-generation sequencing (NGS) and, when available, whole exome sequencing (WES), must be performed as a collaborative effort between the geneticist and the clinical immunologist. Genetic testing represents the gold standard for diagnosing IEIwA [13].

## PRECISION MEDICINE PERSPECTIVE

Overall, the approach to IEIwA treatment consists in multidisciplinary care involving at least the following experts: immunologist, allergist, and eventually a hematologist experienced in HSCT [15,45^▪^] (Table 1).

**Table 1:** Summary of the evidence about currently available tailored treatments for inborn errors of immunity with atopic phenotypes (IEIwA)

IEIwA	Targeted therapy (potential indications)	References
HIES		
AD-HIES STAT3 deficiency (Job syndrome)	Dupilumab (skin manifestations, EoE)	[46–51]
	Omalizumab (skin manifestations – both atopic dermatitis and recurrent infections-, pulmonary manifestations)	[53–57]
IL6ST deficiency	JAK inhibitors: ruxolitinib and tofacitinib (autoinflammation and immune dysregulation manifestations)	[72]
DOCK8 deficiency	Dupilumab, Omalizumab (skin manifestations as bridge to HSCT)	[45^▪^,^61^]
ZNF341 deficiency	Dupilumab (skin manifestations)	[63]
PGM3 deficiency	N-acetylglucosamine and uridine oral supplementation (clinical trial ongoing)	[46]
Comel-Netherton syndrome	Kallikrein specific small-molecules inhibitor (skin manifestations)	[63]
	Gene therapy – lentiviral vector	[64,65]
	Dupilumab (skin manifestations)	[66–69]
	Omalizumab	[70]
	Ustekinumab	[71]
	Anti-IL17 agents – ixekizumab and secukinumab-, TNF-α inhibitors, and anakinra (skin manifestations)	[45^▪^,^72^]
STAT6 GOF	Ruxolitinib or tofacitinib (allergic dysregulation, skin disease/atopic dermatitis)	[27,28^▪▪^]
	Dupilumab (allergic dysregulation atopic dermatitis and asthma)	
WAS and WASL conditions		
WAS	Anakinra (auto-inflammatory manifestations)	[15,75]
	Omalizumab (diffuse pruritic eczema resistant to conventional systemic immunosuppressive therapy)	[76]
ARPC1B deficiency	Dupilumab (atopic dermatitis/eczema)	[78]
IPEX and IPEX-like conditions		
IPEX	Dupilumab (skin manifestations)	[80]
	Gene therapy	[3^▪^,^81^]
CBM-opathies		
CADINS	Dupilumab (allergic dysregulation)	[15,45^▪^,^82^]
	Mepolizumab (allergic dysregulation)	[15]
CARD14 GOF	Ustekinumab (CARD14-associated papulosquamous eruption, namely psoriasis and pityriasis rubra pilaris)	[45^▪^,^83^,^84^]
Miscellaneous		
JAK1 GOF	JAK inhibitor – baricitinib and upadacitinib (atopic dermatitis, chronic inflammatory rheumatism, or inflammatory bowel diseases)	[86]
STAT1 GOF	Ruxolitinib (cytopenia, T1DM); baricitinib and upadacitinib (autoimmune and autoinflammatory manifestations)	[3^▪^,^86^]
OS (associated with multiple genetic abnormalities)	Dupilumab (bridge to HSCT)	[45^▪^]

AD, autosomal dominant; ARPC1B, actin related protein 2/3 complex subunit 1B; CADINS, CARD11-associated atopy with dominant interference of NF-κB signaling; CARD, Caspase Recruitment Domain; CBM, CARD Family Member – B cell chronic lymphocytic leukemia/lymphoma 10 (BCL10) – mucosa-associated lymphoid tissue lymphoma translocation gene 1 (MALT1); DOCK8, Dedicator of cytokinesis 8; EoE, Eosinophilic Esophagitis; GOF, gain-of-function; HIES, hyperIgE syndrome; HSCT, hematopoietic stem-cells transplantation; IL, interleukin; IL6ST, Interleukin 6 (IL6) Signal Transducer; IPEX, immune dysregulation, polyendocrinopathy, enteropathy, X-linked; JAK, Janus-kinase; OS, Omenn syndrome; PGM3, phosphoglucomutase 3; STAT, signal transducer and activator of transcription; T1DM, type 1 diabetes mellitus; TNF, Tumor necrosis factor; WAS, Wiskott–Aldrich Syndrome; WASL, WAS-like; ZNF341, zinc finger protein 341.

### Hyper-immunoglobulin E syndromes

*STAT3-AD-HIES*: Apart from prophylactic therapy based on antimicrobial agents (i.e., antistaphylococcal and antifungal agents) and topical antiseptics, which reduce the risk of cutaneous and sinopulmonary bacterial infections [18], nowadays tailored treatments are also available. Dupilumab was generally thought to be effective considering the increased IL-4 expression. Indeed, the positive outcome of this treatment, with substantial improvement of the cutaneous lesions, pruritus and IgE levels, has been confirmed in many reports [46–49]. Moreover, dupilumab may improve EoE signs and symptoms [50,51]. As noticed in several patients, the use of omalizumab in *STAT3*-AD-HIES leads to the improvement of skin signs and symptoms, the decrease of serum IgE and the improvement of pulmonary manifestations [52]. In combination with co-trimoxazole and inhaled tobramycin, no recurrent pulmonary or skin infection and a considerable improvement in skin lesions have been reported as well [53–57]. The role of HSCT is still being studied, but some encouraging reports seem to show an improvement in immunological reconstitution and nonimmunologic features (skin and pulmonary signs and symptoms). Nonetheless, previous controversial reports of transplant failure and death should be considered, too [58]. Harrison *et al.* recently reported a 100% survival rate, minimal peri-transplant complications, improved infection rates, resolution of skin disease, and stabilization/improvement of lung function, both from a clinical and radiological perspective. Immunologically, serum IgE decreased, and normalization of Th17-secreting IL-17 was demonstrated. Yet, the effects on nonimmunological manifestations, such as connective tissue diseases, are still to be confirmed, which makes HSCT controversial in HIES [59]. HSCT may be considered in specific cases, mainly based on infectious manifestations, patient age, and quality of life.

*DOCK8 AR-HIES*: HSCT is the only recommended curative option. To ensure the best survival rates and reduction of complications, it must be performed at the early stages of the disease. After HSCT, patients showed a remarkable improvement in lymphocyte function, with reduced IgE levels and better T and B cell function, and an improvement of clinical manifestations, with infections and eczema resolved more quickly than food allergies [60]. Biological drugs can be used as bridge therapy to HSCT to improve the patient's quality of life. To date, reports show that, with dupilumab or omalizumab, interesting steps forward have been taken in terms of skin lesions [44,61].

*Other HIES*: As a result of dupilumab administration, significant clinical improvement and reduced IgE level were reported in a *ZNF341* deficient adult patient with severe atopic dermatitis [62]. Considering *PGM3* deficiency, only little evidence is available: the positive outcome of HSCT has been reported as successful in two out of three patients treated. The use of N-acetylglucosamine and uridine oral supplementation is still under assessment [44]. Other treatment options for *SPINK5* deficient patients include kallikrein inhibitors (protein replacement therapy) [63], gene therapy (lentiviral vector encoding the *SPINK5* gene under investigation) [64,65], dupilumab (a randomized clinical trial is under recruitment) [66–69], omalizumab [70], ustekinumab [71], anti-IL17 agents (ixekizumab and secukinumab), TNF-α inhibitors, and anakinra [44,72]. Moreover, constitutive STAT3 phosphorylation caused by IL6ST mutation makes inhibition with JAK inhibitors (ruxolitinib or tofacitinib) an effective treatment option in these patients [73] Overall, JAK inhibition may be a future treatment option also for STAT3 DN [74]. Considering *STAT6* GOF, therapeutic approaches include JAK inhibitors (ruxolitinib and tofacitinib) and dupilumab. Clinical improvement was achieved with both classes of drugs in patients not responding to corticosteroids, topical tacrolimus, oral methotrexate, and mepolizumab [28^▪▪^,^40^].

### Wiskott–Aldrich syndrome and Wiskott–Aldrich syndrome-like conditions

*WAS*: apart from topical emollients, topical corticosteroids and, as proposed in some studies, antiseptic baths for dermatitis, antimicrobial prophylaxis, and immunoglobulin replacement therapy (IgRT) are considered as the pillars of supportive therapy with HSCT and gene therapy as curative treatment [15,75]. Conventional immunosuppressive drugs (corticosteroids, cyclosporine) are usually administered to control immune dysregulation clinical manifestations. Some studies have shown the effectiveness of biological agents: anakinra to treat auto-inflammatory manifestations and omalizumab to treat diffuse pruritic eczema resistant to conventional systemic immunosuppressive therapy [76].

*ARPC1B:* autoinflammatory manifestations could be kept under control by immunosuppressive therapy such as corticosteroids, mofetil mycophenolate, and rapamycin. The use of anti-TNF drugs has given unsatisfying results. To date, HSCT is the only curative treatment with a significant survival rate and limited posttransplant morbidity [77]. In case of atopic disorders, the use of biological drugs targeting Th2 signaling (i.e., dupilumab) may be considered [78].

### Immune dysregulation, polyendocrinopathy, enteropathy, X-linked and immune dysregulation, polyendocrinopathy, enteropathy, X-linked-like conditions

The use of tacrolimus or rapamycin on the mTOR pathway to inhibit effector T cells proliferation could alleviate the signs and symptoms of the disease by reducing the ratio between effector T cells and Treg cells. Unfortunately, on the other hand, the risk of infections increases, too. Therefore, allo-HSCT is the best treatment option, but the transplant must be performed before the development of organ damage [43,79]. Rituximab combined with other immunosuppressive drugs may be an effective option. Furthermore, dupilumab resulted effective in treating eczema [80]. Moreover, gene therapy restores protein function through lentivirus-mediated *FOXP3* gene transfer to CD4^+^ T, reinstating Tregs function [3^▪^,^81^].

### CMB-opathies

*CADINS*: depending on the patient's immune profile and infectious history, antimicrobial prophylaxis and IgRT might be a viable treatment option. Due to Th2/Th1 imbalance, potential targeted therapies against allergic immune dysregulation, such as dupilumab or mepolizumab, could also be considered. Finally, promising *in vitro* results were achieved with glutamine, thanks to its immunomodulatory functions through immune metabolism modulations, as it partially restores T-cell proliferation [15,44,82].

*MALT1 deficiency*: To date, HSCT has been successfully used to treat this condition [44].

*CARD14 GOF*: Ustekinumab has been successfully used in patients carrying CARD14 GOF mutations, as reports have demonstrated an increased production of IL-23 and IL-17/IL-22 by dendritic cells and T cells [43,83,84].

### Miscellaneous

Early HSCT is the only curative option for OS [85]. Targeted therapies downregulating Th2 response are considered as new and safe candidates for OS management; yet, recent *in vitro* data have shown only a mild reduction of IL-4 production after dupilumab treatment vs. control [44]. The refraction of autoimmune and autoinflammatory manifestations of *STAT1* GOF and *STAT3* GOF to common immunosuppressants, even combined, makes their treatment quite complex. Ruxolitinib normalizes Th17 and Treg lymphocyte counts and reduces hyperresponsiveness to IFN. Recently, ruxolitinib has been used to treat several cases of *STAT1*-GOF and *STAT3*-GOF with cytopenia. In addition, ruxolitinib was able to induce remission of T1D in a patient with *STAT1*-GOF [3^▪^]. JAK inhibitor (baricitinib and upadacitinib) successfully kept *JAK1* GOF disease under control [86].

## CONCLUSION

IEIwA are a complex and heterogeneous group of conditions that challenge conventional diagnostic and therapeutic paradigms. It is fundamental to distinguish these rare monogenic disorders from more common multifactorial atopic diseases. The diagnostic approach has radically changed thanks to the recent advances, enabling precise identification of the underlying genetic defects and facilitating tailored treatment strategies.

The current therapeutic options for IEIwA highlight the need for a multidisciplinary approach that combines targeted biological therapies with traditional antimicrobial prophylaxis and, in some cases, HSCT. Nonetheless, the safety and effectiveness of these therapies in the long run are still being studied.

Further research is necessary to clarify the pathogenetic mechanisms behind IEIwA, improve treatment protocols and explore new therapeutic targets. Once these knowledge voids are filled, clinicians will be able to further tailor interventions, achieve more satisfactory outcomes and improve patients’ quality of life. Indeed, the precision medicine approach represents a unique opportunity to transform the care of patients with IEIwA, narrowing the gap between rare monogenic diseases and more common allergic disorders with multifactorial origins.

## Acknowledgements

*None.*

### Financial support and sponsorship

*None.*

### Conflicts of interest

*M.G. reports personal fees from Sanofi. The other authors declare that they have no conflict of interests to disclose in relation to this paper.*

## REFERENCES AND RECOMMENDED READING

Papers of particular interest, published within the annual period of review, have been highlighted as:

▪ of special interest
▪▪ of outstanding interest

## References

[1] Delmonte OM Castagnoli R Calzoni E Notarangelo LD. Inborn errors of immunity with immune dysregulation: from bench to bedside. Front Pediatr 2019; 7:353. 31508401 10.3389/fped.2019.00353 PMC6718615

[2] Notarangelo LD Bacchetta R Casanova JL Su HC. Human inborn errors of immunity: an expanding universe. Sci Immunol 2020; 5:eabb1662. 32651211 10.1126/sciimmunol.abb1662 PMC7647049

[3▪] Giardino G Romano R Lougaris V. Immune tolerance breakdown in inborn errors of immunity: paving the way to novel therapeutic approaches. Clin Immunol 2023; 251:109302. 36967025 10.1016/j.clim.2023.109302

[4] El-Sayed ZA El-Ghoneimy DH Ortega-Martell JA. Allergic manifestations of inborn errors of immunity and their impact on the diagnosis: A worldwide study. World Allergy Organ J 2022; 15:100657. 35783543 10.1016/j.waojou.2022.100657 PMC9218584

[5] Lyons JJ Milner JD. Primary atopic disorders. J Exp Med 2018; 215:1009–1022. 29549114 10.1084/jem.20172306 PMC5881472

[6] Milner JD. Primary atopic disorders. Annu Rev Immunol 2020; 38:785–808. 32126183 10.1146/annurev-immunol-042718-041553

[7] Stadler PC Renner ED Milner J Wollenberg A. Inborn error of immunity or atopic dermatitis: when to be concerned and how to investigate. J Allergy Clin Immunol Pract 2021; 9:1501–1507. 33548520 10.1016/j.jaip.2021.01.037

[8] Vaseghi-Shanjani M Smith KL Sara RJ. Inborn errors of immunity manifesting as atopic disorders. J Allergy Clin Immunol 2021; 148:1130–1139. 34428518 10.1016/j.jaci.2021.08.008

[9] Guttman-Yassky E Irvine AD Brunner PM. The role of Janus kinase signaling in the pathology of atopic dermatitis. J Allergy Clin Immunol 2023; 152:1394–1404. 37536511 10.1016/j.jaci.2023.07.010

[10] Horesh ME Martin-Fernandez M Gruber C. Individuals with JAK1 variants are affected by syndromic features encompassing autoimmunity, atopy, colitis, and dermatitis. J Exp Med 2024; 221:e20232387. 38563820 10.1084/jem.20232387 PMC10986756

[11▪▪] Vaseghi-Shanjani M Yousefi P Sharma M. Transcription factor defects in inborn errors of immunity with atopy. Front Allergy 2023; 4:1237852. 37727514 10.3389/falgy.2023.1237852 PMC10505736

[12] Castagnoli R Pala F Bosticardo M. Gut microbiota-host interactions in inborn errors of immunity. Int J Mol Sci 2021; 22:1416. 33572538 10.3390/ijms22031416 PMC7866830

[13] Ozcan E Notarangelo LD Geha RS. Primary immune deficiencies with aberrant IgE production. J Allergy Clin Immunol 2008; 122:1054–1062. 19084106 10.1016/j.jaci.2008.10.023

[14] Niehues T Hardenberg Svon Velleuer E. Rapid identification of primary atopic disorders (PAD) by a clinical landmark-guided, upfront use of genomic sequencing. Allergol Select 2024; 8:304–323. 39381601 10.5414/ALX02520E PMC11460323

[15] Castagnoli R Lougaris V Giardino G. Immunology Task Force of the Italian Society of Pediatric Allergy and Immunology (SIAIP). Inborn errors of immunity with atopic phenotypes: A practical guide for allergists. World Allergy Organ J 2021; 14:100513. 33717395 10.1016/j.waojou.2021.100513 PMC7907539

[16] Cinicola BL Corrente S Castagnoli R. Immunology Task Force of the Italian Society of Pediatric Allergy, Immunology (SIAIP). Primary atopic disorders and chronic skin disease. Pediatr Allergy Immunol 2022; 33: (Suppl 27): 65–68. 35080318 10.1111/pai.13633 PMC9306837

[17] Sams L Wijetilleka S Ponsford M. Atopic manifestations of inborn errors of immunity. Curr Opin Allergy Clin Immunol 2023; 23:478–490. 37755421 10.1097/ACI.0000000000000943 PMC10621644

[18] Bergerson JRE Freeman AF. An update on syndromes with a hyper-IgE phenotype. Immunol Allergy Clin North Am 2019; 39:49–61. 30466772 10.1016/j.iac.2018.08.007

[19] Boos AC Hagl B Schlesinger A. Atopic dermatitis, STAT3- and DOCK8-hyper-IgE syndromes differ in IgE-based sensitization pattern. Allergy 2014; 69:943–953. 24898675 10.1111/all.12416

[20] Hagl B Heinz V Schlesinger A. Key findings to expedite the diagnosis of hyper-IgE syndromes in infants and young children. Pediatr Allergy Immunol 2016; 27:177–184. 26592211 10.1111/pai.12512

[21] Schwerd T Twigg SRF Aschenbrenner D. A biallelic mutation in IL6ST encoding the GP130 co-receptor causes immunodeficiency and craniosynostosis. J Exp Med 2017; 214:2547–2562. 28747427 10.1084/jem.20161810 PMC5584118

[22] Zhang Q Davis JC Lamborn IT. Combined immunodeficiency associated with DOCK8 mutations. N Engl J Med 2009; 361:2046–2055. 19776401 10.1056/NEJMoa0905506 PMC2965730

[23] Minegishi Y Saito M Morio T. Human tyrosine kinase 2 deficiency reveals its requisite roles in multiple cytokine signals involved in innate and acquired immunity. Immunity 2006; 25:745–755. 17088085 10.1016/j.immuni.2006.09.009

[24] Lyons JJ Liu Y Ma CA. ERBIN deficiency links STAT3 and TGF-β pathway defects with atopy in humans. J Exp Med 2017; 214:669–680. 28126831 10.1084/jem.20161435 PMC5339676

[25] Renner ED Hartl D Rylaarsdam S. Comèl-Netherton syndrome defined as primary immunodeficiency. J Allergy Clin Immunol 2009; 124:536–543. 19683336 10.1016/j.jaci.2009.06.009 PMC3685174

[26] Zhang Y Yu X Ichikawa M. Autosomal recessive phosphoglucomutase 3 (PGM3) mutations link glycosylation defects to atopy, immune deficiency, autoimmunity, and neurocognitive impairment. J Allergy Clin Immunol 2014; 133:1400–1409. e5. 24589341 10.1016/j.jaci.2014.02.013 PMC4016982

[27] Minskaia E Maimaris J Jenkins P. Autosomal dominant STAT6 gain of function causes severe atopy associated with lymphoma. J Clin Immunol 2023; 43:1611–1622. 37316763 10.1007/s10875-023-01530-7 PMC10499697

[28▪▪] Baris S Benamar M Chen Q. Severe allergic dysregulation due to a gain of function mutation in the transcription factor STAT6. J Allergy Clin Immunol 2023; 152:182–194.e7. 36758835 10.1016/j.jaci.2023.01.023 PMC10330134

[29▪▪] Sharma M Leung D Momenilandi M. Human germline heterozygous gain-of-function STAT6 variants cause severe allergic disease. J Exp Med 2023; 220:e20221755. 36884218 10.1084/jem.20221755 PMC10037107

[30] Brigida I Zoccolillo M Cicalese MP. T-cell defects in patients with ARPC1B germline mutations account for combined immunodeficiency. Blood 2018; 132:2362–2374. 30254128 10.1182/blood-2018-07-863431 PMC6265646

[31] Lam MT Coppola S Krumbach OHF. A novel disorder involving dyshematopoiesis, inflammation, and HLH due to aberrant CDC42 function. J Exp Med 2019; 216:2778–2799. 31601675 10.1084/jem.20190147 PMC6888978

[32] Bacchetta R Barzaghi F Roncarolo MG. From IPEX syndrome to FOXP3 mutation: a lesson on immune dysregulation. Ann N Y Acad Sci 2018; 1417:5–22. 26918796 10.1111/nyas.13011

[33] Verbsky JW Chatila TA. Immune dysregulation, polyendocrinopathy, enteropathy, X-linked (IPEX) and IPEX-related disorders: an evolving web of heritable autoimmune diseases. Curr Opin Pediatr 2013; 25:708–714. 24240290 10.1097/MOP.0000000000000029 PMC4047515

[34] Goudy K Aydin D Barzaghi F. Human IL2RA null mutation mediates immunodeficiency with lymphoproliferation and autoimmunity. Clin Immunol 2013; 146:248–261. 23416241 10.1016/j.clim.2013.01.004 PMC3594590

[35] Biggs CM Lu HY Turvey SE. Monogenic immune disorders and severe atopic disease. Nat Genet 2017; 49:1162–1163. 28747751 10.1038/ng.3925

[36] Dorjbal B Stinson JR Ma CA. Hypomorphic caspase activation and recruitment domain 11 (CARD11) mutations associated with diverse immunologic phenotypes with or without atopic disease. J Allergy Clin Immunol 2019; 143:1482–1495. 30170123 10.1016/j.jaci.2018.08.013 PMC6395549

[37] Peled A Sarig O Sun G. Loss-of-function mutations in caspase recruitment domain- containing protein 14 (CARD14) are associated with a severe variant of atopic dermatitis. J Allergy Clin Immunol 2019; 143:173–181. e10. 30248356 10.1016/j.jaci.2018.09.002

[38] McKinnon ML Rozmus J Fung SY. Combined immunodeficiency associated with homozygous MALT1 mutations. J Allergy Clin Immunol 2014; 133:1458–1462. e7. 24332264 10.1016/j.jaci.2013.10.045

[39] Villa A Notarangelo LD Roifman CM. Omenn syndrome: inflammation in leaky severe combined immunodeficiency. J Allergy Clin Immunol 2008; 122:1082–1086. 18992930 10.1016/j.jaci.2008.09.037

[40] Wang Y Ma CS Ling Y. Dual T cell– and B cell–intrinsic deficiency in humans with biallelic RLTPR mutations. J Exp Med 2016; 213:2413–2435. 27647349 10.1084/jem.20160576 PMC5068239

[41] Del Bel KL Ragotte RJ Saferali A. JAK1 gain-of-function causes an autosomal dominant immune dysregulatory and hypereosinophilic syndrome. J Allergy Clin Immunol 2017; 139:2016–2020. e5. 28111307 10.1016/j.jaci.2016.12.957

[42] Ma CA Xi L Cauff B. Somatic STAT5b gain-of-function mutations in early onset nonclonal eosinophilia, urticaria, dermatitis, and diarrhea. Blood 2017; 129:650–653. 27956386 10.1182/blood-2016-09-737817 PMC5290989

[43] Ponsford MJ Klocperk A Pulvirenti F. Hyper-IgE in the allergy clinic––when is it primary immunodeficiency? Allergy 2018; 73:2122–2136. 30043993 10.1111/all.13578

[44] Criado PR Miot HA Ianhez M. Eosinophilia and elevated IgE serum levels: a red flag: when your diagnosis is not a common atopic eczema or common allergy. Inflamm Res 2023; 72:541–551. 36637497 10.1007/s00011-023-01690-7

[45▪] Giancotta C Colantoni N Pacillo L. Tailored treatments in inborn errors of immunity associated with atopy (IEIs-A) with skin involvement. Front Pediatr 2023; 11:1129249. 37033173 10.3389/fped.2023.1129249 PMC10073443

[46] Sogkas G Hirsch S Jablonka A. Dupilumab to treat severe atopic dermatitis in autosomal dominant hyper-IgE syndrome. Clin Immunol 2020; 215:108452. 32360519 10.1016/j.clim.2020.108452

[47] Staudacher O Krüger R Kölsch U. Relieving job: Dupilumab in autosomal dominant STAT3 hyper-IgE syndrome. J Allergy Clin Immunol Pract 2022; 10:349–351. e1. 34536614 10.1016/j.jaip.2021.08.042

[48] Matucci-Cerinic C Viglizzo G Pastorino C. Remission of eczema and recovery of Th1 polarization following treatment with Dupilumab in STAT3 hyper IgE syndrome. Pediatr Allergy Immunol 2022; 33:e13770. 35470938 10.1111/pai.13770 PMC9321118

[49] Wang HJ Yang TT Lan CCe. Dupilumab treatment of eczema in a child with STAT3 hyper-immunoglobulin E syndrome. J Eur Acad Dermatol Venereol 2022; 36:e367–e369. 34927771 10.1111/jdv.17889

[50] Dixit C Thatayatikom A Pappa H Knutsen AP. Treatment of severe atopic dermatitis and eosinophilic esophagitis with dupilumab in a 14-year-old boy with autosomal dominant hyper-IgE syndrome. J Allergy Clin Immunol Prac 2021; 9:4167–4169. 10.1016/j.jaip.2021.06.049 34280586

[51] Votto M Naso M Brambilla I. Eosinophilic gastrointestinal diseases in inborn errors of immunity. J Clin Med 2023; 12:514. 36675441 10.3390/jcm12020514 PMC9867405

[52] Licari A Castagnoli R Panfili E. An update on anti-IgE therapy in pediatric respiratory diseases. Curr Respir Med Rev 2017; 13:22–29. 29290750 10.2174/1573398X13666170616110738 PMC5735517

[53] Gomes N Miranda J Lopes S. Omalizumab in the treatment of hyper-IgE syndrome: 2 case reports. J Investig Allergol Clin Immunol 2020; 30:191–192. 10.18176/jiaci.0469 31820738

[54] Bard S Paravisini A Avilés-Izquierdo JA. Eczematous dermatitis in the setting of hyper-IgE syndrome successfully treated with omalizumab. Arch Dermatol 2008; 144:1662–1663. 19075161 10.1001/archdermatol.2008.510

[55] Alonso-Bello CD Jiménez-Martínez M del C Vargas-Camaño ME. Partial and transient clinical response to omalizumab in IL-21-induced low STAT3-phosphorylation on hyper-IgE syndrome. Case Rep Immunol 2019; 2019:6357256. 10.1155/2019/6357256 PMC6637684 31355024

[56] Marcotte GV. Omalizumab therapy for hyper-IgE syndrome. J Allergy Clin Immunol 2008; 121: (Suppl 1): S88.

[57] Papaioannou O Karampitsakos T Katsaras M. Clinical improvement in job syndrome following administration of co-trimoxazole, omalizumab and inhaled tobramycin. Adv Respir Med 2021; 89:585–588. 10.5603/ARM.a2021.0079 34668182

[58] Castagnoli R Delmonte OM Calzoni E Notarangelo LD. Hematopoietic stem cell transplantation in primary immunodeficiency diseases: current status and future perspectives. Front Pediatr 2019; 7:295. 31440487 10.3389/fped.2019.00295 PMC6694735

[59] Harrison SC Tsilifis C Slatter MA. hematopoietic stem cell transplantation resolves the immune deficit associated with STAT3-dominant-negative hyper-IgE syndrome. J Clin Immunol 2021; 41:934–943. 33523338 10.1007/s10875-021-00971-2 PMC8249289

[60] Aydin SE Freeman AF Al-Herz W. Hematopoietic stem cell transplantation as treatment for patients with DOCK8 deficiency. J Allergy Clin Immunol Pract 2019; 7:848–855. 30391550 10.1016/j.jaip.2018.10.035 PMC6771433

[61] Ollech A Mashiah J Lev A. Treatment options for DOCK8 deficiency-related severe dermatitis. J Dermatol 2021; 48:1386–1393. 34043252 10.1111/1346-8138.15955

[62] Lévy R Béziat V Barbieux C. Efficacy of dupilumab for controlling severe atopic dermatitis in a patient with hyper-IgE syndrome. J Clin Immunol 2020; 40:418–420. 31993867 10.1007/s10875-020-00751-4 PMC9642001

[63] Liddle J Beneton V Benson M. A potent and selective kallikrein-5 inhibitor delivers high pharmacological activity in skin from patients with netherton syndrome. J Invest Dermatol 2021; 141:2272–2279. 33744298 10.1016/j.jid.2021.01.029

[64] Di WL Larcher F Semenova E. Ex-vivo gene therapy restores LEKTI activity and corrects the architecture of netherton syndrome-derived skin grafts. Mol Ther 2011; 19:408–416. 20877344 10.1038/mt.2010.201 PMC3034839

[65] Di WL Mellerio JE Bernadis C. Phase I study protocol for ex vivo lentiviral gene therapy for the inherited skin disease, netherton syndrome. Hum Gene Ther Clin Dev 2013; 24:182–190. 24329107 10.1089/humc.2013.195

[66] Süßmuth K Traupe H Loser K. Response to dupilumab in two children with Netherton syndrome: Improvement of pruritus and scaling. J Eur Acad Dermatol Venereol 2021; 35:e152–e155. 32810299 10.1111/jdv.16883

[67] Murase C Takeichi T Taki T. Successful dupilumab treatment for ichthyotic and atopic features of Netherton syndrome. J Dermatol Sci 2021; 102:126–129. 33773888 10.1016/j.jdermsci.2021.03.003

[68] Steuer AB Cohen DE. Treatment of Netherton syndrome with dupilumab. JAMA Dermatol 2020; 156:350–351. 31995125 10.1001/jamadermatol.2019.4608

[69] Andreasen TH Karstensen HG Duno M. Successful treatment with dupilumab of an adult with Netherton syndrome. Clin Exp Dermatol 2020; 45:915–917. 32472705 10.1111/ced.14317

[70] Yalcin AD. A case of netherton syndrome: successful treatment with omalizumab and pulse prednisolone and its effects on cytokines and immunoglobulin levels. Immunopharmacol Immunotoxicol 2016; 38:162–166. 26592187 10.3109/08923973.2015.1115518

[71] Volc S Maier L Gritsch A. Successful treatment of Netherton syndrome with ustekinumab in a 15-year-old girl. Br J Dermatol 2020; 183:165–167. 31977080 10.1111/bjd.18892

[72] Ragamin A Nouwen AEM Dalm VASH. Treatment experiences with intravenous immunoglobulins, ixekizumab, dupilumab, and anakinra in Netherton syndrome: a case series. Dermatology 2023; 239:72–80. 35998563 10.1159/000525987 PMC9909717

[73] Materna-Kiryluk A Pollak A Gawalski K. Mosaic IL6ST variant inducing constitutive GP130 cytokine receptor signaling as a cause of neonatal onset immunodeficiency with autoinflammation and dysmorphy. Hum Mol Genet 2021; 30:226–233. 33517393 10.1093/hmg/ddab035

[74] Lobo PB Guisado-Hernández P Villaoslada I. Ex vivo effect of JAK inhibition on JAK-STAT1 pathway hyperactivation in patients with dominant-negative STAT3 mutations. J Clin Immunol 2022; 42:1193–1204. 35507130 10.1007/s10875-022-01273-x

[75] Brigida I Scaramuzza S Lazarevic D. A novel genomic inversion in Wiskott-Aldrich–associated autoinflammation. J Allergy Clin Immunol 2016; 138:619–622. e7. 27113846 10.1016/j.jaci.2016.03.007 PMC4969072

[76] Gupta P Subburaj K Jindal A. Atypical Wiskott–Aldrich syndrome without thrombocytopenia partially responding to omalizumab therapy. Clin Exp Dermatol 2022; 47:1013–1016. 35249234 10.1111/ced.15119

[77] Giardino S Volpi S Lucioni F. Hematopoietic stem cell transplantation in ARPC1B deficiency. J Clin Immunol 2022; 42:1535–1544. 35767111 10.1007/s10875-022-01305-6

[78] Chiriaco M Ursu GM Amodio D. Radiosensitivity in patients affected by ARPC1B deficiency: a new disease trait? Front Immunol 2022; 13:919237. 35967303 10.3389/fimmu.2022.919237 PMC9372879

[79] Gambineri E Torgerson TR Ochs HD. Immune dysregulation, polyendocrinopathy, enteropathy, and X-linked inheritance (IPEX), a syndrome of systemic autoimmunity caused by mutations of FOXP3, a critical regulator of T-cell homeostasis. Curr Opin Rheumatol 2003; 15:430–435. 12819471 10.1097/00002281-200307000-00010

[80] Maher MC Hall EM Horii KA. Generalized eczematous dermatitis and pruritus responsive to dupilumab in a patient with immune dysregulation, polyendocrinopathy, enteropathy, X-linked (IPEX) syndrome. Pediatr Dermatol 2021; 38:1370–1371. 34272772 10.1111/pde.14717

[81] Schmetterer KG Haiderer D Leb-Reichl VM. Bet v 1–specific T-cell receptor/forkhead box protein 3 transgenic T cells suppress Bet v 1–specific T-cell effector function in an activation-dependent manner. J Allergy Clin Immunol 2011; 127:238–245. e3. 21211658 10.1016/j.jaci.2010.10.023

[82] Charvet E Bourrat E Hickman G. Efficacy of dupilumab for controlling severe atopic dermatitis with dominant-negative CARD11 variant. Clin Exp Dermatol 2021; 46:1334–1335. 33864281 10.1111/ced.14686

[83] Eytan O Sarig O Sprecher E van Steensel MAM. Clinical response to ustekinumab in familial pityriasis rubra pilaris caused by a novel mutation in CARD14. Br J Dermatol 2014; 171:420–422. 24641799 10.1111/bjd.12952

[84] Kiszewski AE De Almeida HL Jr. Successful treatment with ustekinumab in CARD14-associated papulosquamous eruption in a Brazilian child. Dermatol Ther 2022; 35:e15939. 36239488 10.1111/dth.15939

[85] Mazzolari E Moshous D Forino C. Hematopoietic stem cell transplantation in Omenn syndrome: a single-center experience. Bone Marrow Transplant 2005; 36:107–114. 15908971 10.1038/sj.bmt.1705017

[86] Fayand A Hentgen V Posseme C. Successful treatment of JAK1-associated inflammatory disease. J Allergy Clin Immunol 2023; 152:972–983. 37343845 10.1016/j.jaci.2023.06.004

